# Implantable Cardioverter Defibrillator for Primary Prevention in Children With Arrhythmogenic Right Ventricular Cardiomyopathy: A Case Series

**DOI:** 10.7759/cureus.77253

**Published:** 2025-01-10

**Authors:** Ayako Nagashima-Otsuki, Taku Ishii, Yohei Yamaguchi, Susumu Hosokawa, Shouzaburoh Doi

**Affiliations:** 1 Department of Pediatrics, Institute of Science Tokyo, Tokyo, JPN; 2 Department of Pediatrics, Ota General Hospital, Kawasaki, JPN; 3 Department of Pediatrics, Musashino Red Cross Hospital, Tokyo, JPN; 4 Department of Pediatrics, Tokyo Healthcare University, Tokyo, JPN

**Keywords:** arrhythmogenic right ventricular cardiomyopathy, implantable cardioverter defibrillator, primary prevention, sudden cardiac death, ventricular arrhythmia

## Abstract

Children with arrhythmogenic right ventricular cardiomyopathy (ARVC) are at high risk for sudden cardiac death secondary to arrhythmia. However, indications for implantable cardioverter defibrillators (ICDs) for primary prevention in children with ARVC are unclear. We present three cases of childhood-onset ARVC and discuss the indications of ICD for primary prevention. Case 1 is a 23-year-old woman who was diagnosed with ARVC at the age of 13 years with heart failure. ICD was placed for asymptomatic nonsustained ventricular tachycardia (NSVT) in recent years. Case 2 is an 18-year-old girl who was diagnosed at the age of 14 years with exertional chest pain and biventricular heart failure, which progressively worsened. She was registered for heart transplantation, and a cardiac resynchronization therapy defibrillator was implanted because of sinus bradycardia. Case 3 is an 18-year-old boy who had bigeminal premature ventricular contractions (PVCs) during a heart disease screening in school at the age of 12 years. Although cardiac function was preserved, he had multifocal PVCs and NSVTs even at rest. Although the PVC and NSVT events did not increase after restricting exercise and β-blocker administration, ventricular fibrillation developed at the age of 15 years. Therefore, the ICD was implanted. Based on these cases, the severity of arrhythmia did not necessarily correspond with the right ventricular function in patients with ARVC. Thus, the risk of fatal arrhythmia should be continuously assessed to determine the appropriate timing of ICD placement for primary prevention.

## Introduction

Arrhythmogenic right ventricular cardiomyopathy (ARVC) is an inheritable cardiomyopathy. It is characterized by ventricular arrhythmia (VA), increased risk of sudden cardiac death (SCD), and ventricular dysfunction, which starts from the right ventricle (RV) and eventually extends to the left ventricle [1,2]. ARVC treatment aims to prevent SCD and delay heart failure progression, given the higher SCD risk in younger patients [3,4].

In general, ICD is strongly recommended for patients who have experienced cardiac arrest due to VA as secondary prevention [2]. For those with sustained ventricular tachycardia (VT) and severe cardiac dysfunction, ICD implantation is also recommended as primary prevention according to recent consensus statements or guidelines [2,5-7]. However, there are no clear indications for ICD implantation in patients with nonsustained ventricular tachycardia (NSVT) or moderate cardiac dysfunction, and decisions regarding ICD implantation are typically made by the attending physician on a case-by-case basis [2,5-7]. These days, advancements in genetics have been made in this field, but not all genetic mutations responsible for VA and SCD have been fully elucidated [6,8,9]. Particularly in children, faster heart rates may lead to inappropriate shocks [10]. Additionally, the physical growth of children must be considered when selecting devices [10]. Therefore, extra caution is required when considering ICD implantation as primary prevention in pediatric cases. At the same time, children with ARVC are at a higher risk of VA compared to adults [4]. Therefore, deciding on ICD implantation as primary prevention in children involves complex and nuanced judgment.

Herein, we present three cases of childhood-onset ARVC and examine the risk of SCD in each case to highlight the importance of careful and repeated assessments for the indications of implantable cardioverter defibrillators (ICDs).

## Case presentation

Case 1

**Figure 1:**
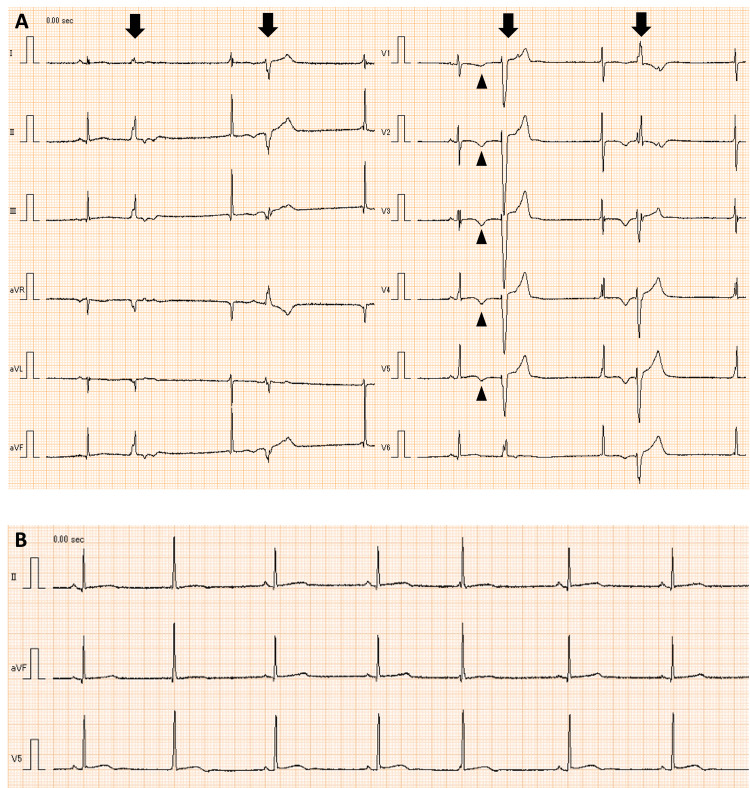
Electrocardiogram at rest of case 1 at the age of 16 years (i.e., three years from diagnosis) A) Negative T waves from V1 to V5 (triangles) and multifocal premature ventricular contractions (bold arrows); B) Sinus bradycardia with a heart rate of 44 beats per minute and junctional rhythm

**Figure 2:**
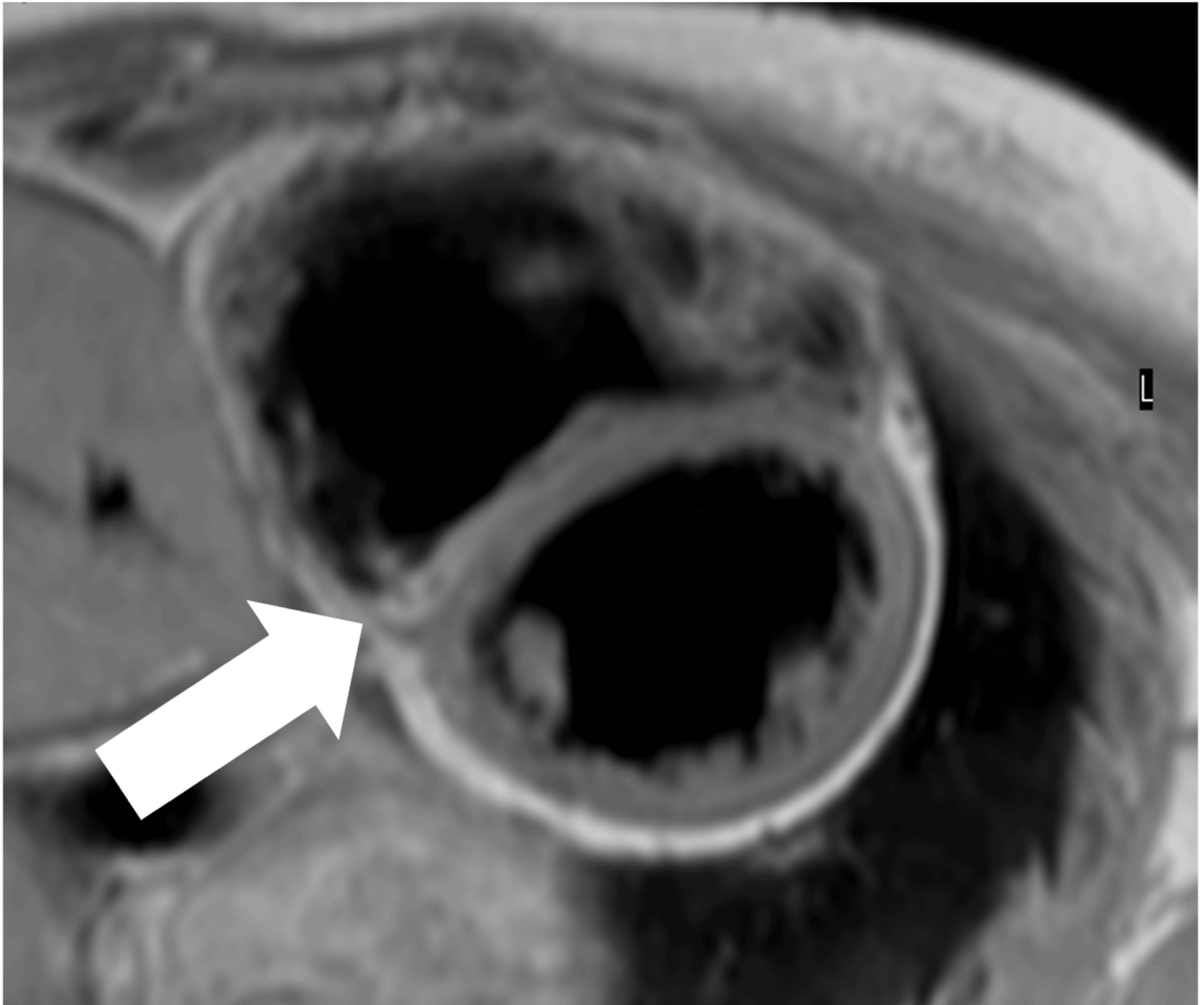
Cardiac magnetic resonance imaging of case 1 at diagnosis T1-weighted magnetic resonance imaging showing fatty replacement of the myocardium of the right ventricle (bold arrow)

A 23-year-old woman started to have frequent chest pain episodes during sleep at the age of 13 years. Her mother had a history of definite ARVC. The electrocardiogram showed bradycardia and premature ventricular contractions (PVCs) with the right bundle branch block (Figure 1). Single-photon emission computed tomographic thallium imaging showed myocardial damage at the apical and inferior walls of the LV. Therefore, cardiac magnetic resonance imaging (MRI) was performed, and fatty tissue replacement of the cardiomyocytes was found at the lateral RV on late gadolinium enhancement and T1-weighted image (Figure 2). Furthermore, moderate biventricular dysfunction was found in cardiac catheterization by measuring the left and the right atrial pressure and calculating EF based on ventricular angiography of the LV and the RV, and cardiac tissue fibrosis was seen on biopsy. Therefore, she was diagnosed with ARVC. No genetic testing has been performed because the patient and her family did not consent.

A β-blocker and angiotensin-converting enzyme inhibitors (ACEis) were initiated; however, her heart failure worsened, and the PVCs became more frequent. In recent years, asymptomatic NSVT, with a VT rate of 132 beats per minute (bpm), was noted. Therefore, ICD was implanted at the age of 22 years. She has not so far received an ICD shock by the age of 23 years.

Case 2

**Figure 3:**
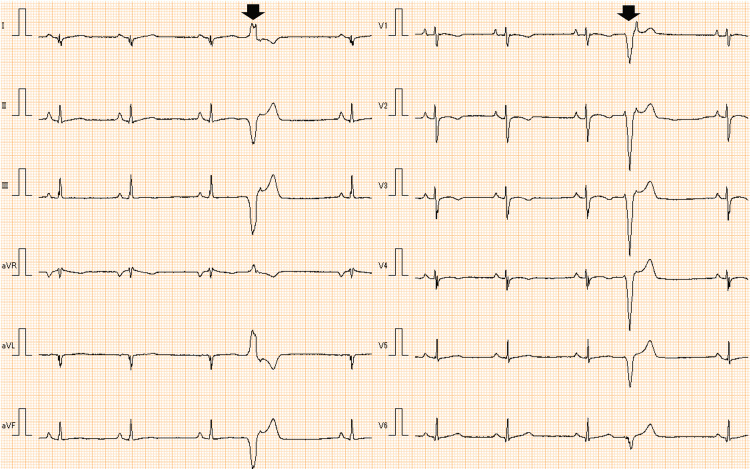
Electrocardiogram at rest of case 2 at diagnosis 12-lead electrocardiogram showing bradycardia, premature ventricular contractions (PVCs) (bold arrows), and low voltage in all leads

**Figure 4:**
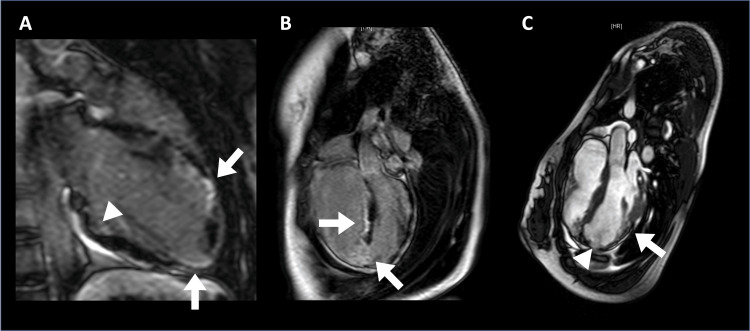
Cardiac magnetic resonance imaging of case 2 at diagnosis Left ventricular ejection fraction, 38%; cardiac index, 1.2 L/min/m² A) Late gadolinium enhancement image showing fibrosis and fatty replacement of the myocardium of the right ventricle (RV) with intense signal at the anterior wall and apex (bold arrows). The area pointed to by a triangle shows thinning of the myocardium of the RV; B) Late gadolinium enhancement image showing fibrosis and fatty infiltration of the myocardium also in the area including the apex and the interventricular septum (bold arrow); C) Sequence of gradient echo cine showing thinning of the myocardium of the area including the posterior wall (bold arrow) and apex (triangle) of the left ventricle. Aneurysm formation is also seen at the apex of the left ventricle (triangle).

An 18-year-old girl first complained of exertional chest pain and dyspnea at the age of 14 years. The electrocardiogram showed bradycardia, PVCs, and low voltage both in precordial leads and in limb leads (Figure 3). The echocardiogram showed deteriorated cardiac function of both the LV and RV. We suspected cardiomyopathy and performed a cardiac MRI, and we found diffuse late gadolinium enhancement, sporadic thinning of the cardiac muscle (Figure 4), and biventricular dysfunction. Furthermore, diffuse myocardial fibrosis and adipose tissue infiltration were revealed on cardiac biopsy. Therefore, she was diagnosed with ARVC. She only had monofocal PVCs as the arrhythmic events. Genetic testing was performed, and a homozygous missense mutation of DSG2 was found.

With an RVEF of 37% by catheterization and an LVEF of 38% by cardiac MRI at the diagnosis, she was subsequently registered with the Japan Organ Transplantation Network for heart transplantation because of worsening heart failure despite medication therapy, including β-blockers, ACEis, and diuretics. A cardiac resynchronization therapy defibrillator (CRT-D) was implanted to address LV systolic failure and prevent cardiac events, particularly those that can be triggered by VAs.

Case 3

**Figure 5:**
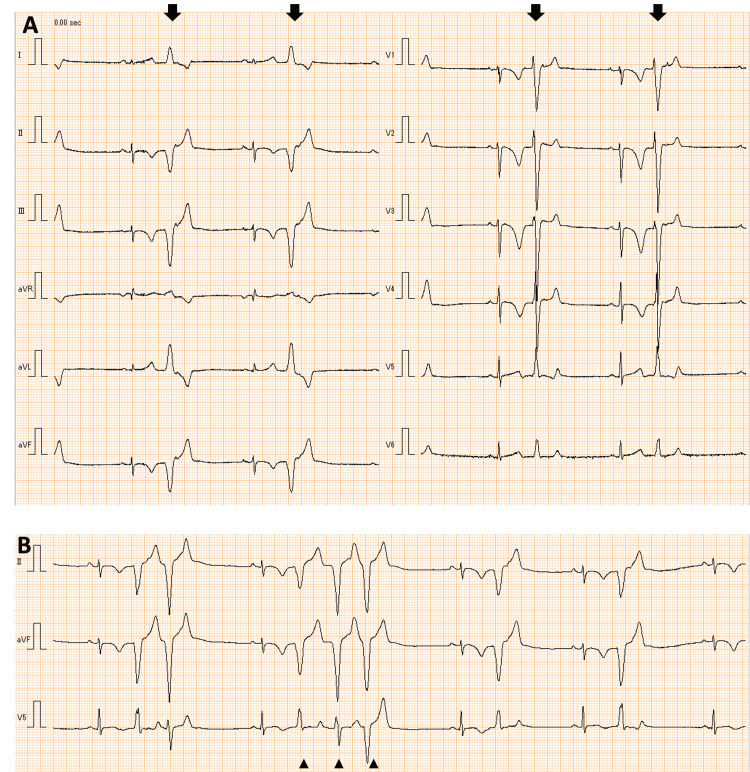
Electrocardiogram of case 3 at rest at the age of 13 years A) Bigeminal premature ventricular contractions (PVCs) (bold arrows); B) Multifocal premature ventricular contractions and non-sustained ventricular tachycardias (triangles)

**Figure 6:**
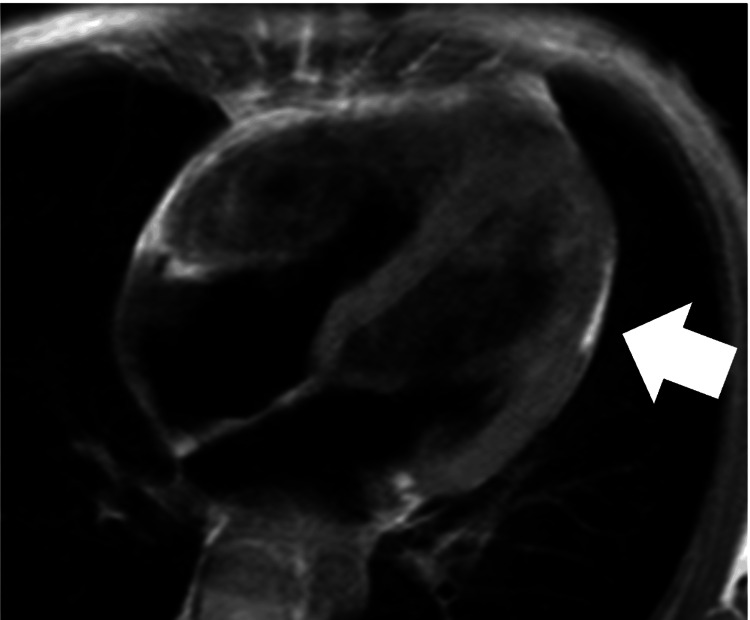
Cardiac magnetic resonance imaging of case 3 at diagnosis T1-weighted magnetic resonance imaging showing fat deposit from anterolateral to the apical wall of the left ventricle

An 18-year-old boy was found to have asymptomatic bigeminal PVCs during a heart disease screening in school at the age of 12 years (Figure 5A). Despite preserved ventricular function, he was subsequently diagnosed with ARVC based on an increase in the number of PVCs with exercise and cardiac MRI findings of fat replacement of the myocardium on the LV apex (Figure 6). Moreover, he had multifocal PVCs and NSVTs (in triplets, with a maximum rate of 182 bpm) even at rest (Figure 5B). No genetic testing has been performed according to the patient’s and the family’s wishes.

Although the PVCs and NSVTs did not become more frequent after exercise restrictions and β-blocker administration, ventricular fibrillation (VF) occurred, which resulted in cardiopulmonary arrest (CPA) during a badminton game at the age of 14 years. He was resuscitated by percutaneous cardiopulmonary support and recovered without neurological sequelae. At the age of 15 years, an ICD was implanted for secondary prevention, and antiarrhythmic drug therapy was reinforced by increasing the β-blocker dose and adding potassium channel blockers and ACEis. At the age of 16 years, he experienced VT at a rate of 250-260 bpm with palpitations and received an appropriate ICD intervention.

The characteristics of the three cases are summarized in Table 1.

**Table 1:** Characteristics of the three cases of ARVC ARVC: arrhythmogenic right ventricular cardiomyopathy; ATP: antitachycardia pacing; CRT-D: cardiac resynchronization therapy defibrillator; CL: cycle length; ICD: implantable cardioverter defibrillator; LBBB: left bundle branch block; LVEF: left ventricular ejection fraction; MRI: magnetic resonance imaging; NSVT: nonsustained ventricular tachycardia; PVC: premature ventricular contraction; RV: right ventricle; RVEF: right ventricular ejection fraction; SCD: sudden cardiac death; THB: total heartbeats; VT: ventricular tachycardia

Cases			Case 1	Case 2	Case 3
Sex			Female	Female	Male
Current age (years)			23	18	18
Age at onset (years)			13	14	12
Age at ICD placement (years)			22	17 (CRT-D)	15
Task Force criteria fulfillment			Major criteria: RVEF 9.7% by MRI, history of ARVC in a first-degree relative	Major criteria: Regional RV dyskinesia in right ventriculography, residual myocytes <60%, inverted T waves in right precordial leads (V1-V4)	Major criteria: Inverted T waves in right precordial leads (V1-V4), NSVT of LBBB morphology
			Minor criterion: 4206 PVCs/24 h	Minor criterion: Late potentials by signal-averaged electrocardiograph in three parameters	Minor criterion: RVEF 41% by MRI
Level of confidence in the diagnosis			Definite ARVC	Definite ARVC	Definite ARVC
Genotype			Not tested	DSG2	Not tested
History of syncope at onset			No	No	No
Family history of ARVC or SCD			Mother (definite ARVC)	No	No
Symptoms	At onset		Chest pain	Chest pain and dyspnea	PVCs during heart disease screening in school
	At ICD placement		Fatigability	Fatigability	Palpitation
Cardiac function	At onset		RVEF 38.9% (catheterization), LVEF 53.2% (catheterization)	RVEF 37% (catheterization), LVEF 38% (MRI)	RVEF 41% (MRI), LVEF 63% (MRI)
	At ICD placement		RVEF 29.2% (catheterization), LVEF 36.8% (catheterization)	Not tested	Not tested
Arrhythmias	At onset	PVC/form	4206 beats/24 h (4.5% of THB), multiform	388 beats/24 h (0.46% of THB), triplet	19501 beats/24 h (17.5% of THB), multiform
		NSVT/VT rate	No	Triplet	Six consecutive beats, maximum VT rate of 182
	At ICD placement	PVC/form	7244 beats/24 h (8.4% of THB), multiform	425 beats/24 h (0.5% of THB), multiform	20233 beats/24 h (20.1% of THB), multiform
		NSVT/VT rate	Triplet, maximum VT rate 128	No	four consecutive beats, maximum VT rate of 214
Ventricular fibrillation			No	No	Age 14
Appropriate ICD interventions			No	Not known	Age 15, VT (CL 250-260 ms) terminated by ATP

## Discussion

Herein, we share our experience on the three cases of childhood-onset ARVC with different clinical courses. Since ARVC diagnosis, cases 1 and 2 had symptoms of heart failure mainly and no arrhythmic events. Conversely, case 3 had no heart failure symptoms but had remarkable arrhythmias, which resulted in CPA. Our discussion on ICD indications in young patients with ARVC was based on these three cases.

In general, ICD implantation is highly recommended for secondary prevention of all cases and primary prevention in cases with severe risk of VA. The International Task Force (ITF) consensus statement categorizes patients who have experienced an aborted SCD secondary to VF, those with sustained VT, and those who have severe cardiac dysfunction as high risk and recommends ICD implantation for those patients [2]. However, the indications for ICD implantation for primary prevention in intermediate-risk cases are controversial. ITF categorizes those who have experienced syncope, NSVTs, and moderate cardiac dysfunction as intermediate-risk cases [2]. The ITF proposes making individualized decisions to implant ICDs in intermediate-risk cases considering the overall clinical profile and individual conditions, such as cultural background or availability. Similar to the ITF, other criteria such as the Padua Criteria 2020, the European Society of Cardiology (ESC), and the group consisting of the American Heart Association (AHA), the American College of Cardiology (ACC), and the Heart Rhythm Society (HRS) suggest nearly the same indications for ICD implantation as primary prevention [5-7]. These indications remain controversial despite the importance of evaluating VA risk when making appropriate judgments for ICD implantation as primary prevention. In children, making a clear indication for ICD implantation for primary prevention is even more difficult because ICD implantation has problems specific to children [10]. However, ICD for primary prevention in ARVC cases is more important for children than for adults because young age is a risk factor for VAs and SCD [4,11-13].

Table 2 summarizes the VA risks of our three cases according to the two risk prediction models (Cadrin-Tourigny et al. and Carrick et al.) and the ICD recommendation from the three guidelines (CS of ITF, the ESC guideline, and the AHA/ACC/HRS guideline) [2,6,7,13,14]. The risk prediction models were derived from the data of adult patients with ARVC (38.2 ± 15.5 years in the study by Cadrin-Tourigny et al., 37 ± 15.1 years in the study by Carrick); however, we applied these models to our patients as a reference because no measure quantifies the VA risk in pediatric patients with ARVC. The risk varied among cases according to the risk-stratification tool employed. In cases 1 and 3 at diagnosis, nearly the same prediction and recommendation were made, except for the study by Carrick et al.; however, the outcomes were different. The five-year prediction model by Carrick et al. accurately predicted the high VA risk of case 3 at diagnosis. In the model, NSVT and 24-hour PVC count have a large influence on the VA risk [14]. Case 1 had multifocal but infrequent PVCs and few NSVTs at diagnosis. On the contrary, case 3 had frequent multifocal PVCs of up to 19501 beats per 24 h and NSVTs with a maximum VT rate of 182 bpm at ARVC onset. Indeed, polymorphic VT, relatively fast VT, and frequent PVCs have been reported to worsen mortality [15-17]. Therefore, the rate, morphology, and frequency of PVCs must be monitored as SCD predictors.

**Table 2:** The risk of ventricular arrhythmias and ICD recommendation in the 3 cases ACC: American College of Cardiology; AHA: American Heart Association; CS: consensus statement; C-T and B model: prediction model disclosed by Cadrin-Tourigny et al. [13] in 2019; ESC: European Society of Cardiology; HRS: Heart Rhythm Society; ICD: implantable cardioverter defibrillator; ITF: International Task Force; VA: ventricular arrhythmia

Cases and the timing of the evaluation	Case 1: female		Case 2: female		Case 3: male	
	At diagnosis	Currently	At diagnosis	Currently	At diagnosis	Right before CPA
Age (years)	13	23	14	18	12	14
5-year VA risk by C-T and B et al.	40.1%	74.2%	27.2%	28.5%	42.3%	41.9%
5-year VA risk by Carrick et al.	34.6%	57.5%	36.5%	19.8%	82.4%	84.6%
ICD recommendation by CS of ITF	Class IIa	Class I	Class I	Class I	Class IIa	Class IIa
ICD recommendation by ESC guideline	Class IIa	Class IIa	Class IIa	Class IIa	Class IIa	Class IIa
ICD recommendation by AHA/ACC/HRS guideline 2017	Class IIa	Class IIa	Class IIa	Class IIa	Class IIa	Class IIa

Furthermore, the risk for VA or SCD may increase chronologically, particularly in younger patients. In case 1, the VA risk predicted by Cadrin-Tourigny et al. and Carrick et al. increased, and the ICD recommendation based on the ITF consensus statement became class I within eight years of ARVC diagnosis. Recent studies have emphasized the importance of recalculating the VA risks at each follow-up or every year using the most recent set of clinical risk factors [14,18]. Therefore, ICD indications must be examined regularly based on the frequency of PVCs and NSVTs, PVC morphology, and NSVT rates.

These days, some genetic mutations have been identified to be responsible for VA or SCD, and it has been proposed to evaluate the need for ICD using the results of genetic tests in ARVC patients [6,8,9]. This makes genetic testing more important in ARVC, especially in patients who have decreased cardiac functions. However, not all the mutations responsible for VA or SCD have been identified, and genetic tests are not available in all regions of the world. Therefore, it is still significant to evaluate clinically accessible data, such as echocardiograms, 24-hour electrocardiograms, and biomarkers for heart failure such as B-type natriuretic peptide (BNP)/N-terminal pro-B-type natriuretic peptide (NTproBNP), comprehensively and chronologically.

## Conclusions

Determining the indication for ICD implantation in young ARVC patients is challenging based on a single criterion. Genetic information may play a more significant role in guiding ICD implantation as primary prevention in the future. However, genetic testing is not universally accessible across all countries or regions, and some patients may decline testing. Therefore, it remains essential to consider multiple criteria and incorporate detailed information about VAs, including waveform, frequency, and rate. Furthermore, in young ARVC patients, the risk of SCD may increase with growth. Therefore, it is crucial to perform regular longitudinal assessments of sudden death risk and to repeatedly evaluate the appropriateness of ICD implantation over time.
